# Innovative public strategies in response to COVID‐19: A review of practices from China

**DOI:** 10.1002/hcs2.122

**Published:** 2024-12-18

**Authors:** You Wu, Zijian Cao, Jing Yang, Xinran Bi, Weiqing Xiong, Xiaoru Feng, Yue Yan, Zeyu Zhang, Zongjiu Zhang

**Affiliations:** ^1^ School of Healthcare Management, Tsinghua Medicine Tsinghua University Beijing China; ^2^ School of Basic Medical Sciences, Tsinghua Medicine Tsinghua University Beijing China; ^3^ Department of Health Policy and Management, Bloomberg School of Public Health Johns Hopkins University Baltimore Maryland USA; ^4^ School of Biomedical Engineering, Tsinghua Medicine Tsinghua University Beijing China; ^5^ Sir Run Run Shaw Hospital, School of Medicine Zhejiang University Hangzhou Zhejiang China

**Keywords:** COVID‐19, emergency response, health care management, pandemic

## Abstract

The COVID‐19 pandemic presented unparalleled challenges to prompt and adaptive responses from nations worldwide. This review examines China's multifaceted approach to the crisis, focusing on five key areas of response: infrastructure and system design, medical care and treatment, disease prevention and control, economic and social resilience, and China's engagement in global health. This review demonstrates the effectiveness of a top‐down command system at the national level, intersectoral coordination, a legal framework, and public social governance. This study also examines medical care and treatment strategies, highlighting the importance of rapid emergency response, evidence‐based treatment, and well‐planned vaccination rollout. Further discussion on disease prevention and control measures emphasizes the importance of adaptive measures, timely infection control, transmission interruption, population herd immunity, and technology applications. Socioeconomic impact was also assessed, detailing strategies for disease prevention, material supply, livelihood preservation, and social economy revival. Lastly, we examine China's contributions to the global health community, with a focus on knowledge‐sharing, information exchange, and multilateral assistance. While it is true that each nation's response must be tailored to its own context, there are universal lessons to be drawn from China's approach. These insights are pivotal for enhancing global health security, especially as the world navigates evolving health crises.

## Graphical abstract

China's COVID‐19 response: infrastructure, medical care, prevention, resilience, and global health engagement. Highlighted China's top‐down command system, intersectoral coordination, and social governance effectiveness. Focused on coordinated emergency response, evidence‐based treatment, strategic vaccine rollout, and IT support in healthcare.

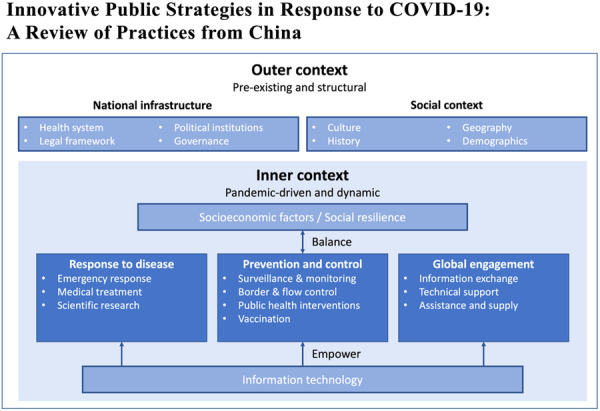

**Abbreviations**
GDP: gross domestic product
PPE: personal protective equipment
SARS: severe acute respiratory syndrome

## 1 INTRODUCTION

The COVID‐19 pandemic has tested the resilience and effectiveness of health systems and emergency responses worldwide [1, 2]. Amidst the complex backdrop of global distress, China's approach to the pandemic stood out not only for its unique methods but also for its efficacy in curtailing the spread of the virus [3, 4], offering valuable lessons in managing such a massive health challenge.

In this review, we aim to elucidate the innovative strategies that contributed to the effectiveness of China's response, highlighting its rapid emergency response, adaptive disease prevention measures, strategic socioeconomic planning, and commitment to global health collaboration. Our objective is two‐fold: first, to meticulously dissect and understand factors that contributed to the effectiveness of China's response, and second, to extrapolate and present insights that can serve as guides for future global responses to health emergencies of a similar magnitude and complexity that resonate beyond borders and timelines.

## 2 MATERIAL AND METHODS

### 2.1 Literature review

A structured search strategy was used across major databases pertaining to COVID‐19 response and management (Appendix method). The search was guided by the 10 pillars proposed by the WHO for the evaluation of COVID‐19 preparedness and response planning [5]. Specific domains were examined, such as theoretical frameworks in emergency management, national infrastructure, medical response, prevention and control strategies, information technology, socioeconomic factors, and global engagement. Within each domain, sub‐categories were defined to aid logic development of the search process. Abstracts of the most relevant papers were thoroughly reviewed, and the additional concepts identified in these abstracts were used to refine and expand the search. We prioritized peer‐reviewed articles, but also covered gray literature such as reports, white papers, and guidelines from trusted organizations and official government‐issued documents or press releases.

### 2.2 Theoretical framework

Over the years, several key theories have been developed to guide emergency management, particularly during a pandemic. In the 1950s, Quarantelli introduced the Theory of Warning and Emergency Response, emphasizing the need for timely responses to emergencies [6]. Later, in the 1980s, Fink proposed the Crisis Management Theory, describing the different stages of crisis [7]. In 1997, Quarantelli proposed 10 criteria for evaluating the management of community disasters, focusing on the impact on social functioning [8]. In 2003, Robert Heath developed the 4R Crisis Management Theory, which covers four critical periods of reduction, readiness, response, and recovery. In 2007, McEntire put forth Strategies and Tactics for Resilience, suggesting the integration of sustainability and emergency management [9]. In 2012, Perry introduced the Protective Action Decision Model, highlighting that emergency management should be a continuous process [10]. In the 2020s, Botzen put forth the Emergency Situation Awareness Theory, highlighting information perception and decision‐making processes during emergencies [11]. The development of these theories reflects the evolving understanding of emergencies over time, providing a theoretical foundation for improving emergency management.

Jung et al. proposed a comprehensive framework to understand the intricacies of national responses to the COVID‐19 pandemic, emphasizing the interplay among drivers, complexities, and uncertainties in handling such health emergencies [12]. Within this framework, pre‐existing and structural systems are viewed as the outer context, and disease prevention and control strategies are viewed as the inner context, thereby capturing the evolving nature of pandemic conditions and the critical nature of evidence‐led responses. Building upon this foundation and contextualizing it for China, our research integrated insights from Wang, who emphasized a scientifically based, full‐cycle crisis management model tailored for infectious diseases [13]. This theory primarily focuses on the response process, including prevention and preparation, investigation and analysis, crisis warning, and response and recovery. Notably, this model accentuates the pivotal role of knowledge‐sharing and information exchange in shaping efficient and adaptable responses to infectious threats.

**Figure 1:**
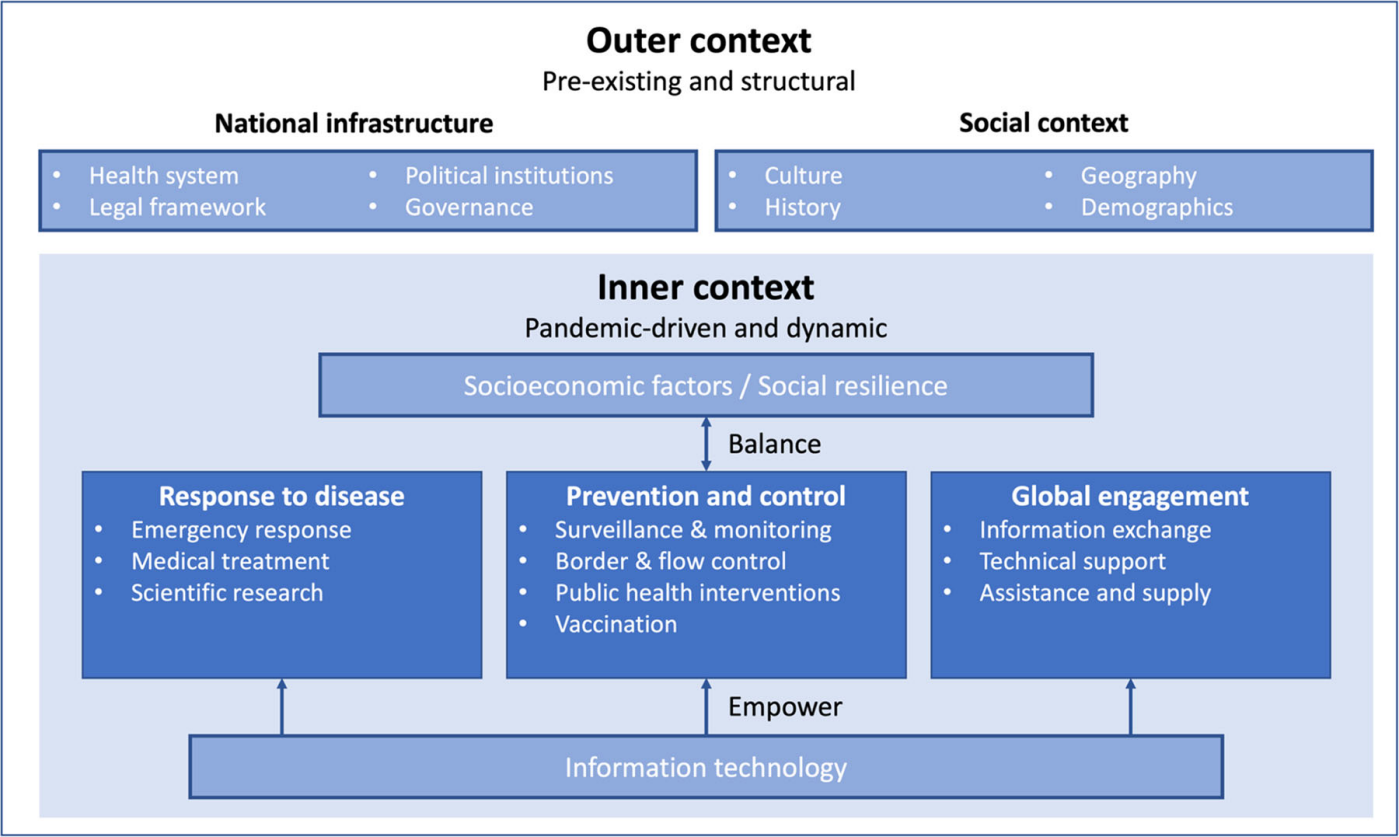
Conceptual framework of emergency responses to COVID‐19.

In our study, we adapt and extend these frameworks into two main realms (Figure 1). The outer context encompasses pre‐existing and structural components like national infrastructure spanning from health systems, legal frameworks, political institutions, and governance, and the social context influenced by culture, history, geography, and demographics. The inner context, which is more pandemic‐driven and dynamic, focuses on China's specific response to the disease, prevention and control measures, and global engagement strategies. Central to our framework is the balancing act between socioeconomic factors and prevention and control efforts, with information technology serving as a powerful catalyst bolstering all aspects of the inner context. Taken together, this review offers an in‐depth examination of China's response strategy to COVID‐19, which should be viewed as coexisting, complementary frameworks that are best used in concert.

## 3 INFRASTRUCTURE AND SYSTEM DESIGN

### 3.1 Global epidemic prevention and control system models

**Figure 2:**
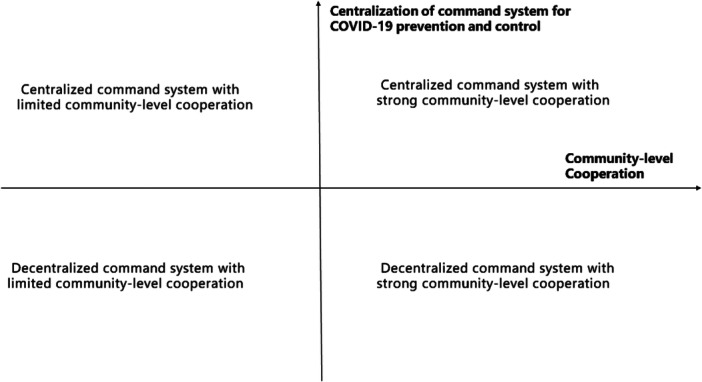
Classification of global epidemic prevention and control systems.

Across the globe, the approach of different countries to pandemic prevention and control are largely shaped by their governance structures and community mobilization capacities. Based on these attributes, we can categorize global epidemic prevention and control systems into four primary models, outlined below and in Figure 2.

The *Decentralized Command System with Limited Community‐Level Cooperation* model is represented by countries such as the United States (US), Germany, and Austria. As federal republics, these nations exhibit regional variations in preventive measures, even in the presence of national guidelines or suggestions [14, 15, 16].

The *Centralized Command System with Limited Community‐Level Cooperation* model is prevalent in some parts of East Asia, Southeast Asia, the Middle East, and North Africa. Although many countries have a unified central command system, it is difficult for community‐level governments to implement policies on time [17, 18]. Factors like resource constraints, organizational issues, and prolonged conflicts in some Middle Eastern and North African areas, have hampered community‐level governance and health care provision [19].

The *Decentralized Command System with Strong Community‐Level Cooperation* model is relatively rare, with Sweden as an example. This model relies on the cooperation of its citizens to effectively control the spread of an epidemic and flatten the curve, thereby alleviating the strains on medical resources [20].

The *Centralized Command System with Strong Community‐Level Cooperation* model is represented by countries like China and Israel [21]. Israel and some Gulf countries have performed relatively well in early containment and preventive measures, followed by extensive vaccination programs [19]. With its centralized command structure, China formulated the “one plan, three systems” policy. This approach, encompassing emergency plans and management systems, has resulted in a successful responded to outbreaks like severe acute respiratory syndrome (SARS), pandemic influenza A (H1N1) pdm09, human infections with avian influenza A (H7N9) virus, and COVID‐19, with the active cooperation of communities and individuals [22, 23].

### 3.2 China's command system for epidemic prevention and control

**Figure 3:**
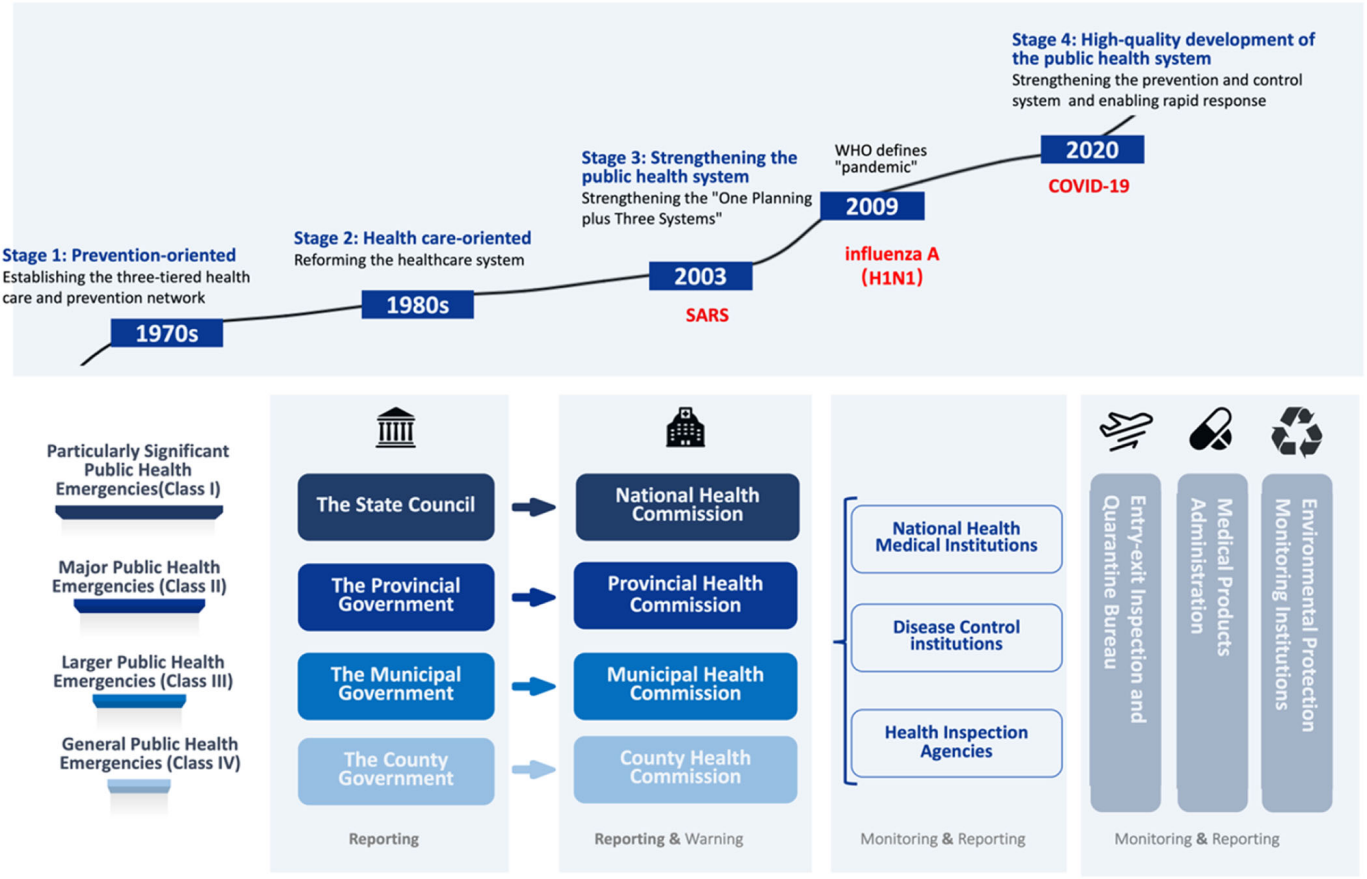
Organizational system of emergency command for public health emergencies in China.

In terms of a vertical chain of command, China's national COVID‐19 prevention and control efforts were led by the Central Committee of the Communist Party of China, with General Secretary Xi Jinping heading the working group. Central and local administrations formed a monitoring and reporting network [24], where provinces, cities, and counties implemented a graded emergency response system across all levels (Figure 3) [25, 26].

In terms of intersectoral coordination, China established a joint prevention and control mechanism in 2009 to combat the global pandemic of H1N1 [27]. Led by the National Health Commission, the mechanism is a platform for coordinating the work of multiple ministries at the central government level.

### 3.3 Public social governance

In prevention and control of the COVID‐19 pandemic, deficiencies in human resources have been a challenging issue faced by many countries [28, 29, 30]. To address this, involving multiple stakeholders in epidemic prevention and control is paramount [31]. Community‐based models globally have yielded important results. For instance, village health volunteers in Thailand, female health workers in Pakistan, and health extension workers in Ethiopia have all had pivotal roles in their respective regions [32]. In particular, China has the strengths of community‐level social governance and has managed to harness the collective power of village committees, community committees, property companies, and volunteers [33]. This strategy combines vertical (top‐down command) and horizontal (geographic grid management) systems to locate the main responsible bodies and establish protective networks for epidemic warning, investigation, monitoring, and surveillance [34], thereby fortifying the effectiveness of COVID‐19 prevention and control efforts [35, 36].

### 3.4 Laws, regulations, and contingency plans

Over the years, China has established a public health legal system based on the Constitution [37, 38] (Appendix Table A1). China issued the “Opinions on Punishing Crimes of Obstructing the Prevention and Control of the Novel Coronavirus Infection Pneumonia,” to strengthen public security and market supervision to ensure social order [39].

In terms of contingency plans, China has established a system of health emergency plans at different administrative levels. Since 2003, timely assessments and evaluations have been carried out for each public health emergency response [40]. Special risk assessments have been routinely conducted for major public health emergencies, natural disasters, accidents, and other large‐scale events [41].

## 4 MEDICAL CARE AND TREATMENT

### 4.1 Emergency response

In the face of COVID‐19 and other outbreaks, as well as other sudden disasters, the US [42, 43], Israel [44, 45] and some European countries [46] have established relatively complete emergency medical systems and mechanisms. When disasters occur, medical personnel, materials and medical resources can be deployed at the first time. China's response to the COVID‐19 outbreak in Wuhan City and Hubei Province was marked by a surge in patient cases and a shortage of medical resources. Over 42,000 medical personnel from 346 national medical teams were summoned and deployed; 16 cities and counties in Hubei Province were directly supported by 19 other provinces and the military system [47]. Over 9,100 critical care beds were converted to treat severe cases, and resources were centralized to provide the best quality of care [48, 49]. For mild cases, a hierarchical prevention and treatment network was established using fangcang shelter hospitals, newly built hospitals, renovated existing hospitals, and requisitioned hotels and sanatoriums, among other facilities, to expand bed capacity in a short period of time [50]. Sixteen fangcang hospitals with more than 14,000 beds were built in 10 days, offering treatment to one‐quarter of Wuhan's COVID‐19 patients [47]. In just over 10 days, two hospitals (Huoshenshan and Leishenshan) were built using modular design [51].

**Figure 4:**
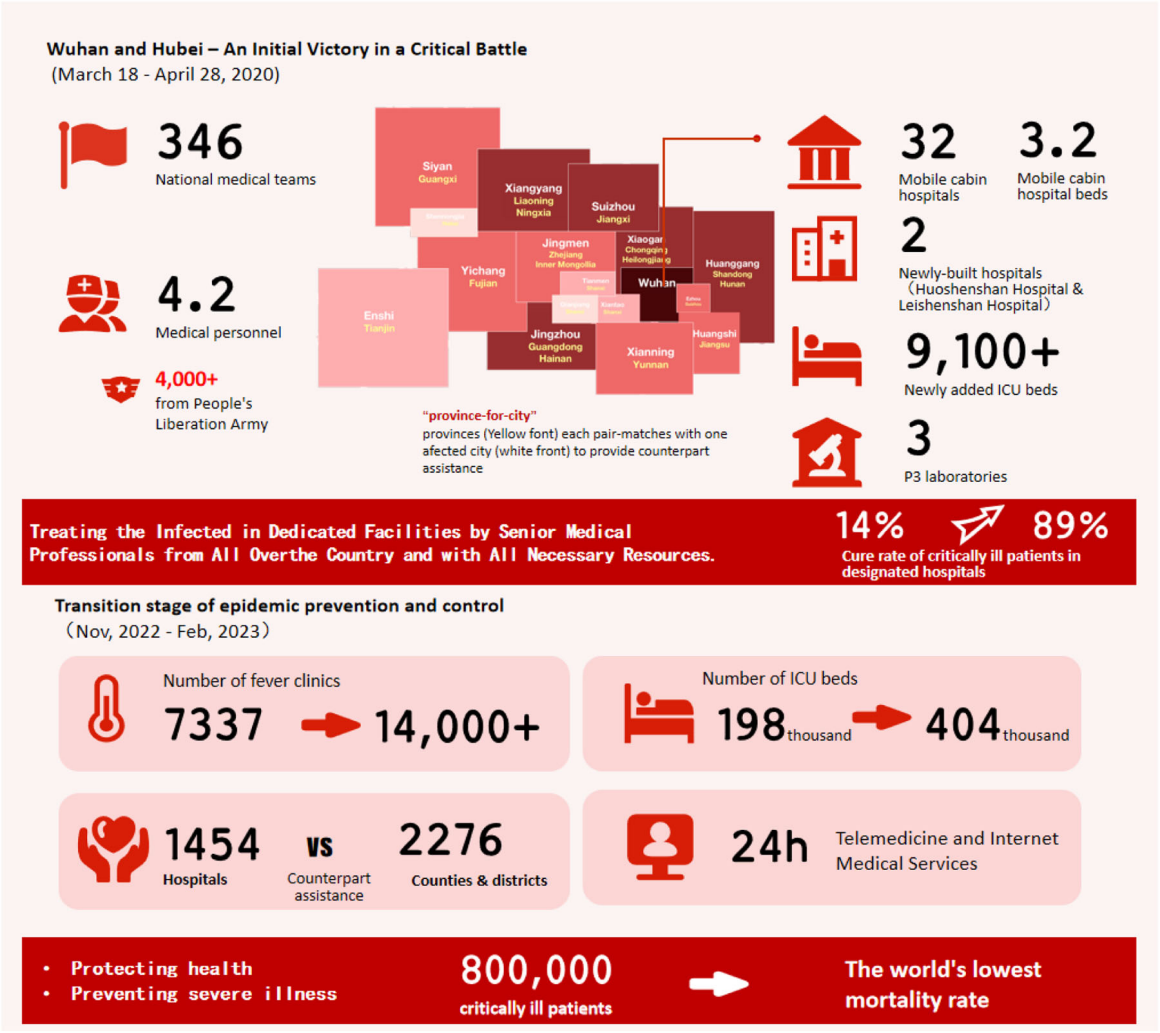
Emergency response and routine disease control of COVID‐19 in China.

The above coordinated emergency response measures led to improvements in patient admission and recovery rates, as well as reductions in infection and mortality rates. The recovery rate for severely ill patients in Wuhan City treated at designated hospitals increased from 14% to 89% [47] (Figure 4).

### 4.2 Medical care

With ongoing changes in the COVID‐19 situation globally, countries continue to adjust their diagnosis and treatment strategies, especially with the emergence and rapid spread of highly transmissive and immune‐evasive Omicron variants, which further accelerated the pace of such adjustments [52, 53, 54]. During the epidemic, China's health system was actively engaged in patient testing [55], contact tracing [56], isolation [57], treatment [58], education, and community management. Treatment and management strategies were continuously adjusted based on virus mutations and development of the epidemic [59]. Patients with suspected COVID‐19 infection were triaged at fever clinics, and severe cases were treated at designated hospitals [60]. Hospitals with respiratory disease treatment capacity were placed on standby [61]. Isolation and treatment capabilities were enhanced by building fangcang field hospitals [62], isolation centers [63], and health stations for high‐risk individuals [64]. Classified treatment involved accurate patient classification, with high‐risk patients treated in designated hospitals, and mild cases and asymptomatic patients admitted to fangcang field hospitals. Post‐recovery, patients were transferred home for self‐isolation [65].

From November 2022 to February 2023, China enacted a series of measures to cope with the peak of infection, including increasing the number of fever clinics in secondary and tertiary medical institutions [66]. expanding the number of beds for severe cases [66] and strengthening the capacity building of primary health‐care institutions [67]. Public hospitals played a crucial role, effectively handling the daily increase of 10,000 patients with severe illness during the peak of the pandemic. During the transition period, nearly 800,000 severely ill patients received effective treatment, with a single‐day peak of 128,000 hospitalized patients with severe illness. Despite these challenges, the COVID‐19 mortality rate in China remained at the lowest level globally [48].

### 4.3 Evidence‐based national guidelines

The National Health Commission of China has disseminated 10 versions of the “Diagnosis and Treatment Protocol for COVID‐19 Patients” to direct clinical diagnosis, management, treatment, and care. Refinements and modifications to the protocol were supported by evidence‐based medicine and progression of the pandemic in China [68]. For instance, the third version introduced nucleic acid testing for diagnosing suspected and confirmed cases [69]. Later, the eighth version established this as the “gold standard” for diagnosis [70]. Owing to the surge in cases during the Omicron outbreak and the constraints in nucleic acid testing capacity, the ninth [65] and tenth versions [71] of the protocol incorporated antigen testing as an auxiliary method. As scientific understanding of COVID‐19 advanced and antiviral drugs became more available, the protocol underwent continual improvements in terms of general treatment and critical care. Traditional Chinese medicine (TCM) has also been incorporated into the treatment of COVID‐19, known as the “China Solution.” In patient care, the signature “three medicines and three prescriptions” approach was recommended by experts alongside nonpharmacological therapies, such as acupuncture [72].

## 5 DISEASE PREVENTION AND CONTROL

### 5.1 Overview of international prevention and control measures

The preventive and control measures adopted by different countries are usually related to their population distribution; geographic location; political, economic, and cultural factors, and their policy implementation capacity [73]. For example, countries with greater health‐care resources tend to implement more preventive and control policies [74]. Countries with more neighboring countries tend to implement more border control policies, and countries with denser populations have adopted mask‐wearing policies [20].

Among all countries, mandatory mask‐wearing, testing and contact tracing, and travel restrictions are associated with more favorable health outcomes during the outbreak. Although these findings are consistent with COVID‐19 mathematical models, the effectiveness of such policies depends on their implementation levels [75].

### 5.2 Adapting prevention and control measures

China has continuously adjusted its prevention and control measures based on virus mutations, epidemic changes and vaccination coverage [76]. China's policies has evolved through three phases: emergency prevention and control, normalized emergency prevention and control, and the transition from normalized emergency prevention and control to regular prevention and control. To ensure a stable transition as the pandemic progressed, the “20‐point measures” and “Additional 10‐point measures” were introduced [77, 78]. These efforts have resulted in the successful management of cluster outbreaks and have ensured a stable transition between phases [79]. By adopting situation‐appropriate prevention and control measures at different stages, China has been able to withstand the impact of multiple global pandemic waves with minimal cost.

### 5.3 Timely control of infection sources

In China, a robust network of nucleic acid and antigen testing capabilities has been established [80, 81]. Innovative methods, such as stratified, block‐based, grid‐based, and rolling nucleic acid screening, have been explored to increase efficiency while minimizing the impact on daily life [82].

A multi‐triggered monitoring system has also been developed, which includes medical institutions [83], fever clinics, pharmacies, and private clinics as “sentry posts” [84].

A large‐scale infectious disease network reporting system has been established. Following the 2003 SARS epidemic, China built a national system in 2004, covering nearly all secondary and higher medical institutions and including 350,000 staff members [85].

Rapid isolation of infection sources and high‐risk individuals has been prioritized. These facilities have implemented centralized and home isolation measures for individuals with COVID‐19 infection or at high risk of infection [86].

Continuous monitoring of variant strains has been conducted. This has enabled a dynamic understanding of genetic variations of SARS‐CoV‐2 and their potential impact on vaccines, drugs, and the overall risk of pandemic prevention and control [87].

### 5.4 Disruption of transmission routes

A closed‐loop management system was established in China to oversee pandemic prevention and control from the perspective of international to domestic scenarios, encompassing departments such as border control and customs, diplomacy, health, transportation, civil aviation, and disease control [88].

Efficient epidemiological investigations have been carried out via individual case investigations and cluster outbreak investigations, which are promptly reported through a network reporting system [89].

Scientific management of mobility has been enacted at both individual and regional levels (Table 1) [90]. And extensive education and promotion have been conducted to encourage individuals to take responsibility for their own health [91].

**Table 1:** COVID‐19 prevention and control strategies during three transitional phases in China [56, 84, 90].

Time periods			20th January 2020 to 10th November 2022	11th November 2022 to 7th January 2023	8th January 2023 to date
Phase			When COVID‐19 was classified as a Class B infectious disease but subject to the preventive and control measures of Class A infectious diseases	Transitional period	When COVID‐19 is managed with measures of Class B infectious diseases
Prevention and Control Policy			Dynamic zero‐Covid policy		Focusing on ensuring public health and preventing severe cases
Timely control of infection sources	Testing and detection	Nucleic acid & antigen testing	(1) Conducting regular nucleic acid testing for the general population (2) Conducting testing for all those in need, especially for at‐risk populations and institutions 2–7 times/week (3) Determining the scope of the population to be tested according to the risk level, and conducting regional nucleic acid testing if necessary (4) Nucleic acid testing as the primary testing method, supplemented by antigen testing	(1) Reducing frequency of nucleic acid testing (2) Increasing use of antigen testing	(1) Detecting infected cases mainly through clinic visits (2) Health monitoring at home (3) Testing of key populations
		Surveillance and early warning systems	(1) Multi‐departments joint prevention and control measures such as ventilation disinfection and body temperature measurement in public gathering places (2) Nucleic acid testing at key stalls in large markets (especially seafood markets) (3) Routine sewage testing (4) Surveillance at sentinel sites such as pharmacies and fever clinics (5) Close monitoring of variant strains		
	Case reporting		Immediate report required for suspected cases, clinically diagnosed cases, confirmed cases, and asymptomatic carriers through the Emergency Public Reporting System (EPRS) within 2 h		Reporting required for suspected cases, clinically diagnosed cases, confirmed cases, and asymptomatic carriers through the Emergency Public Reporting System (EPRS) within 24 h
	Management of people	Confirmed cases	Being transferred to designated medical institutions or fangcang shelter hospitals for medical treatment or applying medical observation in a centralized isolation site	Asymptomatic and mild cases being isolated at home if home‐based protocols were met	(1) Taking necessary treatment and measures to control transmission according to the condition (2) Confirmed cases shall not go out unless necessary
		Suspected cases	Individual isolation and immediate nucleic acid confirmation	Immediate nucleic acid and antigen testing	Voluntary at‐home health monitoring
		Close contact	7 days of quarantine at designated facilities and 3 days of at‐home health monitoring	Gradual transition from 5‐day centralized quarantine and 3‐day home quarantine to 5‐day at‐home or voluntary centralized quarantine	
		Secondary contact	7 days of self‐quarantine at home	No longer identified	
		People from high‐risk areas	7 days of quarantine at designated facilities	7 days of self‐quarantine at home	
		Workers in high‐risk positions exiting closed‐loop operations	7 days of quarantine at designated facilities or at home	5 days of at‐home health monitoring	
	Border control	Inbound flights	(1) Circuit breaker bans for incoming flights (2) Requirement of 2 negative nucleic acid tests within 48 h before boarding	(1) Scraping circuit breaker bans for incoming flights (2) Requirement of 1 negative nucleic acid tests within 48 h before boarding	(1) Closed‐loop transfer and centralized quarantine measures no longer being implemented (2) Incoming personnel being required by customs to fill in a health declaration card ‐ those with normal declarations and no abnormalities in routine quarantine at customs ports being allowed to enter directly
		Inbound arrivals	7 days of quarantine at designated facilities and 3 days of at‐home health monitoring	5 days of quarantine at designated facilities and 3 days of at‐home health monitoring	
		Inbound goods	Performing preventive disinfection to all inbound items, as well as implementing sampling and monitoring of imported cold‐chain products		No longer implementing preventive disinfection to all inbound items nor sampling or monitoring of imported cold chain food
Interruption of transmission routes	Epidemiological investigation		Big data, artificial intelligence and other technologies applied to identify every infected person and track every close contact for quarantine		Precise epidemiological investigations no longer being mandatory
	Management of areas		(1) Classifying risk areas as high, medium or low (2) High‐risk areas: staying at home, door‐to‐door service (3) Medium‐risk areas: staying within the area, avoiding gathering (4) Low‐risk areas: individuals protection, avoiding gathering	(1) The regional classifications adjusted from “high, medium, and low risk” to “high and low risk” to minimize the number of restricted persons (2) High risk areas mostly confined to residential units or blocks, and not being extended at will (3) Mass nucleic acid testing no longer organized at the level of administrative areas	Prevention and control measures focused on critical places, institutions, and groups of people
	Non‐pharmaceutical intervention		Promoting mask wearing, proper social distancing, avoiding crowds, frequent handwashing, and regular ventilation		
Building population immunity barrier	Vaccination		(1) Promoting vaccination among the elderly, people with chronic illnesses, and children (2) Expanding coverage of booster shots and rolling out sequential booster immunization based on technologies different from those used in initial inoculations (3) Continuously promoting vaccine development and optimizing vaccination strategies based on progress of research development and clinical trials		
	Protecting vulnerable groups		Emphasizing on the protection of nursing homes, schools, childcare institutions and medical institutions, etc. Some of these institutions having more stringent restrictions on the mobility of people		Emphasizing on protecting places in which vulnerable people are located, but with reduced mobility restrictions

### 5.5 Building population herd immunity

**Figure 5:**
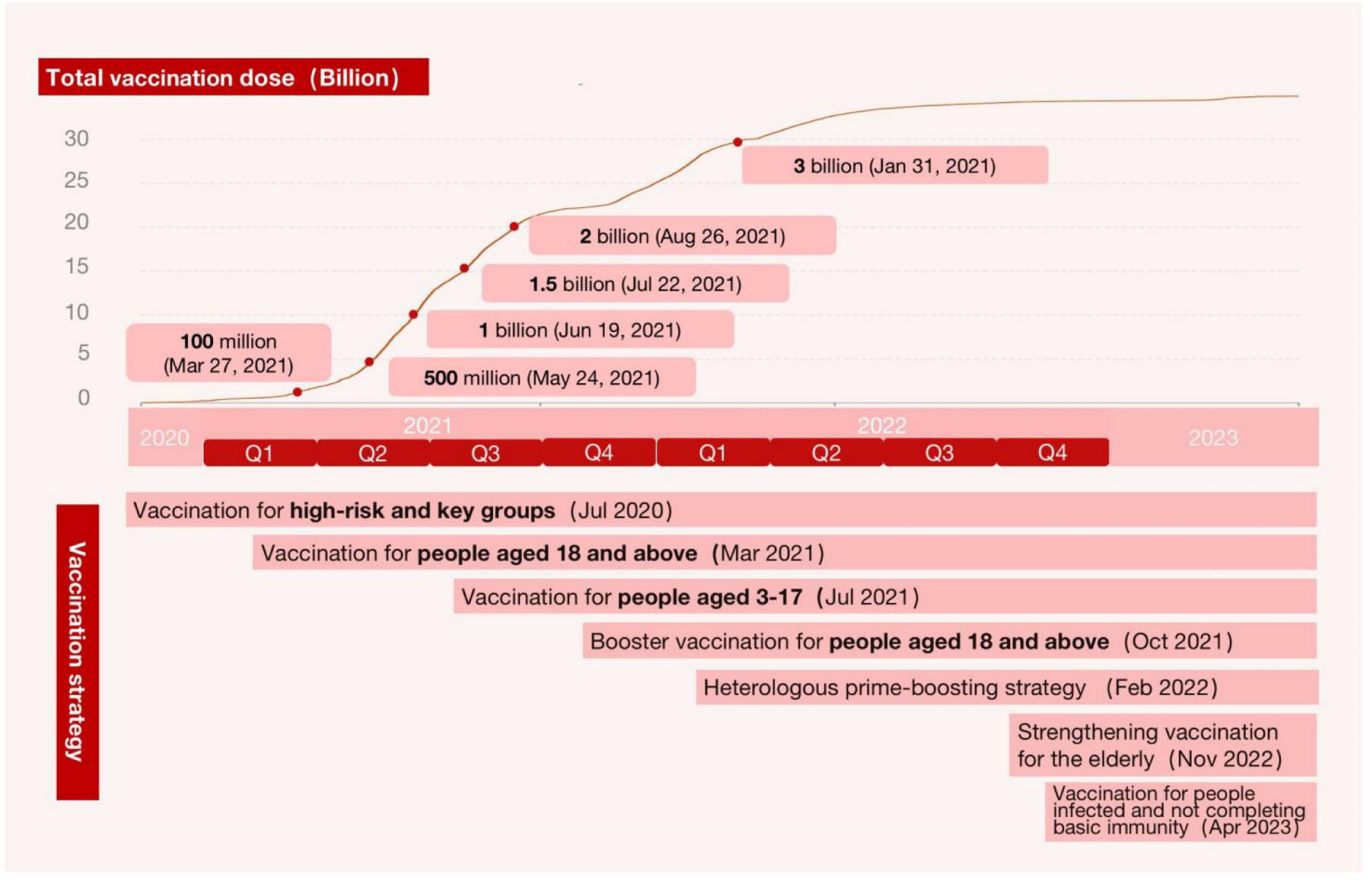
COVID‐19 Vaccination rollout plan.

Since December 2020, vaccinations have been strategically administered in phases to those groups that are most susceptible to infection [92]. To continuously monitor the gap between establishing an immunity barrier and addressing vaccine hesitancy among key groups [93], China has implemented incentives, improved the multi‐level adverse reaction reporting system, and instituted a compensation plan in case of adverse events [94] (Figure 5).

Postvaccination safety and efficacy tracking surveys have taken place to evaluate the immunity barrier in the Chinese population. Statistics on SARS‐CoV‐2 immunoglobulin (Ig) M and IgG antibody levels among the population have been routinely collected [95].

China has implemented measures to protect vulnerable groups. Local governments have implemented tiered health management based on the level of health risk, utilizing color codes [94].

Efforts have also been made to expedite the development of broad‐spectrum vaccines, multi‐valent vaccines, and new vaccines targeting current and potential epidemic strains (Table 2) [95].

**Table 2:** Timeline of COVID‐19 vaccine and drug development in China.

	2020	2021	2022	2023
Inactivated vaccines	Sinopharm (Beijing) (Jun 30),	Sinovac‐CoronaVac (Feb 15),		
	Sinopharm (Wuhan) (Dec 30)	KCONVAC (May 14),		
		IMBCAMS (Jun 6)		
Adenovirus‐vectored vaccines		Ad5‐nCoV (Intramuscular injection) (Feb 24)	Ad5‐nCoV (aerosolise) (Sep 15)	
Protein subunit vaccines		Zifivax (Mar 18)	Likang V‐01 (Sep 15),	SCTV01E (Mar 22)
			SCTV01C (Dec 4),	
			SCB‐2019 (Dec 4),	
			WestVac Biopharma (Dec 5)	
Vaccines using attenuated influenza virus as vectors			dNS1‐RBD (Dec 5)	
Nucleic acid vaccines				SYS6006 (Mar 23)
Neutralizing antibody		BRII‐196/BRII‐198 (Dec 8)		
Small‐molecular drug			Azovudine approved for the treatment of COVID‐19 infection (Jul 25)	VV116 (Jan 29),
				SIM0417 (Jan 29),
				RAY1216 (Mar 23)

*Note*: Timeline of COVID‐19 vaccine and drug development in China. ^a^Sinopharm (Beijing): Inactivated COVID‐19 Vaccine (Vero Cell), developed by Beijing Institute of Biological Products. ^b^Sinopharm (Wuhan): Inactivated COVID‐19 Vaccine (Vero Cell), developed by Wuhan Institute of Biological Products. ^c^Sinovac‐CoronaVac: Sinovac COVID‐19 vaccine, developed by Sinovac Biotech. ^d^Ad5‐nCoV: Trade‐named Convidecia, developed by CanSino Biologics. ^e^ZF2001: Trade‐named Zifivax or ZF‐UZ‐VAC‐2001, developed by Anhui Zhifei Longcom in collaboration with the Institute of Microbiology at the Chinese Academy of Sciences. ^f^KCONVAC: Minhai COVID‐19 vaccine, developed by Shenzhen Kangtai Biological Products Co., Ltd and its subsidiary, Beijing Minhai Biotechnology Co., Ltd. ^g^IMBCAMS: a COVID‐19 vaccine developed by Institute of Medical Biology, Chinese Academy of Medical Sciences. ^h^Likang V‐01: a COVID‐19 vaccine developed by a subsidiary of Livzon Pharmaceutical Group Inc. ^i^SCB‐2019: a COVID‐19 vaccine developed by Clover Biopharmaceuticals. ^j^SCTV01C: a COVID‐19 vaccine developed by Sinocelltech. ^k^WSK‐V101: a COVID‐19 vaccine candidate developed by WestVac BioPharma Co., Ltd. and West China Hospita, Sichuan University. ^l^dNS1‐RBD: CA4‐dNS1‐nCoV‐RBD, developed by Xiamen University, the University of Hong Kong, and Beijing Wantai Biological Pharmacy Enterprise. ^m^SCTV01E: a COVID‐19 vaccine developed by SinoCellTech. ^n^SYS6006: a SARS‐CoV‐2 mRNA vaccine developed by Zhongqi Pharmaceutical Technology Co., Ltd. of CSPC Pharmaceutical Group. ^o^WSK‐V102C: a trivalent recombinant protein vaccines targeting XBB.1.5 developed by WestVac BioPharma Co., Ltd and West China Hospita, Sichuan University. ^p^BRII‐196 and BRII‐198 are investigational, neutralizing monoclonal antibodies manufactured by Brii Biosciences. ^q^VV116 is an oral drug of nucleoside analog against SARS‐CoV‐2, developed by Junshi Biosciences. ^r^SIM0417 is an oral small‐mol‐ecule antiviral agent that targets the SARS‐CoV‐2 3CLpro, developed by Simcere Pharmaceutical Co., Ltd. ^s^RAY1216 is a novel α‐ketoamide based peptidomimetic inhibitor of SARS‐CoV‐2 main protease, developed by Guangdong Zhongsheng Pharmaceutical Co., Ltd.

### 5.6 Information technology empowerment

#### 5.6.1 Emerging tools

Information technologies have played a pivotal role in the global efforts to prevent and control COVID‐19. These have been instrumental in enhancing diagnostic speed, saving lives, and improving epidemic management [96]. For instance, surveillance cameras and portable digital recorders are used to monitor crowd gatherings in public areas [97], and virtual reality technology aids in physical therapy and cognitive rehabilitation for patients [98]. Furthermore, data science, infectious disease modeling, and digital communication technologies are crucial in the timely detection of infectious disease and control of its spread [99, 100].

Contact tracing is a vital component in containing the spread of infectious diseases. Contact tracing methods can be categorized along two dimensions: individual or group, static or dynamic contact tracing. The evolution of contact tracing technology has transitioned from static individual tracking methods, such as offline and online questionnaires, to dynamic individual tracking methods involving wearable wireless sensors, radio frequency identification (RFID), and global positioning system (GPS) devices. Contact tracing has further advanced to dynamic group tracking using data‐driven and artificial intelligence (AI) technologies [101].

Mobile applications (apps) supporting contact tracing have emerged as dynamic group tracking solutions and are being adopted by governments worldwide to manage COVID‐19. Notable examples include the TraceTogether app in Singapore [102], Stopp Corona app in Austria, SwissCovid app in Switzerland [103], COVID Symptom Tracker app in the US and United Kingdom (UK) [104], and the Health Code app in China.

#### 5.6.2 Practical experience in China

China has effectively applied information technologies to manage the COVID‐19 pandemic, with a focus on clinical medical services (online hospitals and telemedicine) [105], public health responses (digital health codes, travel cards, drones, thermal imaging devices, and facial recognition) [106], and emergency preparedness planning (e‐commerce and logistics) [107]. Advanced technologies including mobile networks, big data, cloud computing, the Internet of Things, AI, and 5th generation mobile communication technology (5G) have provided crucial technical support in China's COVID‐19 response efforts [108].

**Figure 6:**
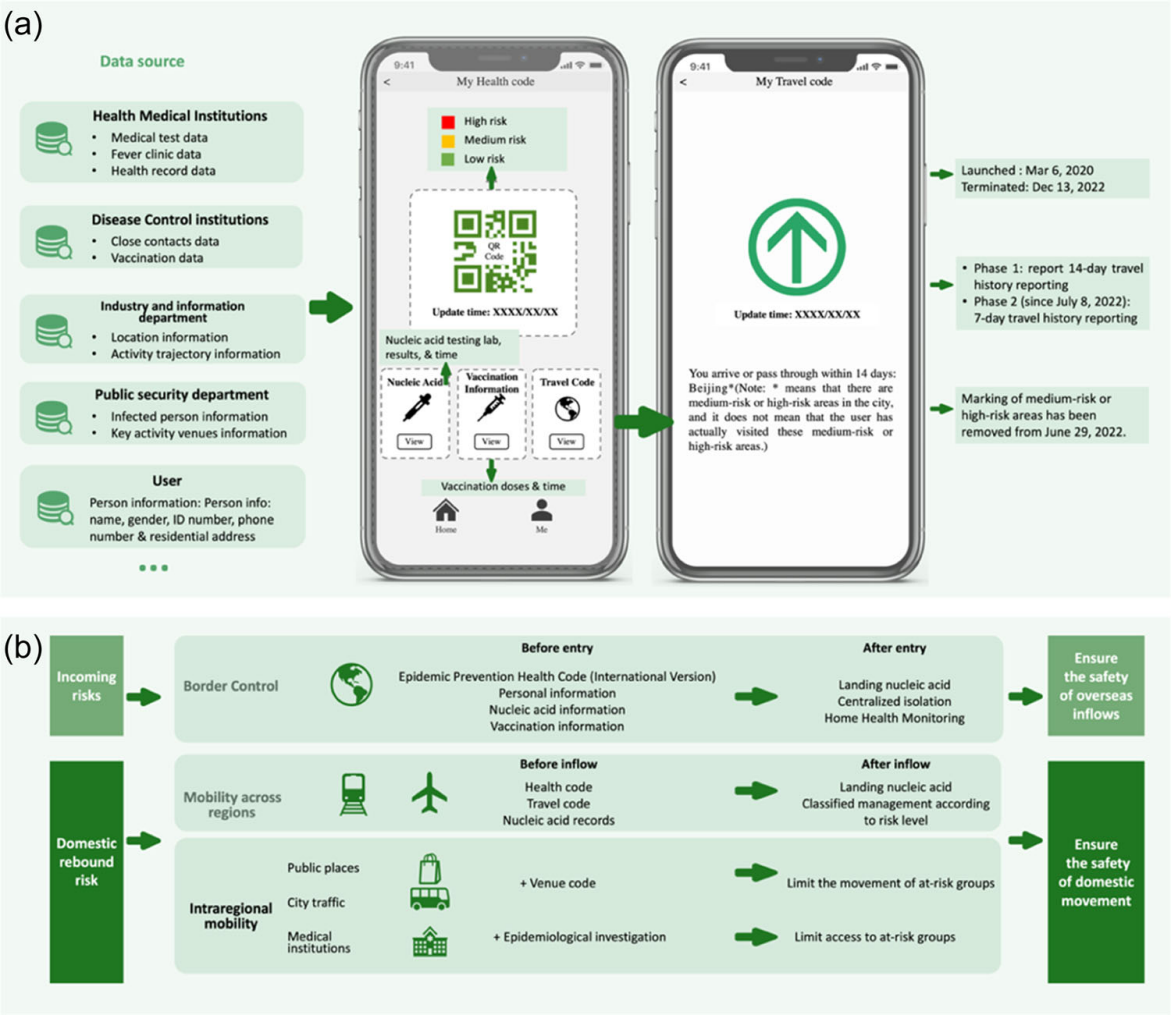
Application of the health code system in COVID‐19 management in China. (a) The user interface of health code and travel code; (b) The use of the code system in managing domestic and international risks.

At the outset of the COVID‐19 outbreak, to enhance contact tracing, isolation, and clinical management efficiency, technology companies such as Tencent and Alibaba partnered with the Chinese government to launch health codes on WeChat and Alipay platforms. Initially implemented in Shenzhen and Hangzhou in February 2020 [109], the health code system was adopted by all 31 provincial administrative regions within 39 days, becoming an indispensable tool for Chinese citizens during the pandemic [110]. The system comprises five categories of information: basic information, health risks, nucleic acid testing, vaccine information, and travel information [111, 112] (Figure 6a). Furthermore, user‐friendly features (“health codes for family” and “health codes of others”) were designed for vulnerable groups such as older people and children.

From April 8, 2020, overseas travelers were required to declare the international version of the health code and present a negative nucleic acid test result before entering China [113]. To detect and intervene in the pandemic, the health code was required in public places or while using public transport. Medical institutions had stricter regulations, requiring patients to report their information and obtain a green flow control code before seeking treatment [114]. For cross‐regional travel, individuals had to self‐declare on the destination city application platform, obtain the local health code, and be assessed for risk level, which determined their entry status and potential isolation management measures [115] (Figure 6b).

#### 5.6.3 Data security for health codes

The health code system in China collected extensive data, including confirmed and suspected cases, close contacts, medical testing (nucleic acid and antibodies), fever clinics, individual locations, area risks, transportation, entry and exit, customs inspection and quarantine, national health records, and self‐reported data [111]. Unlike Google and Apple's Bluetooth‐based contact tracing, China's digital health code system uses algorithms and big data technology for risk assessment [115]. For precise transmission risk assessment, a centralized system was designed to upload data to a central server [112].

To address privacy and security concerns, the National Health Commission of China issued a notice on February 4, 2020 requiring the strengthening of information technology support for COVID‐19 prevention and control, with a focus on personal information protection [116]. Further notices clarified that personal information collected for COVID‐19 prevention and control cannot be used for other purposes [117]. Throughout 2020 and 2021, China issued the National Standard for Information Security Technology‐Personal Information Security Specification (GB/T 35273‐2020) [118], the National Standard for Health and Medical Data Security Guidelines (GB/T 39725‐2020) [119], the Data Security Law of the People's Republic of China [120], and the Personal Information Protection Law of the People's Republic of China [121], reinforcing legal standards for personal information and data security.

## 6 ECONOMIC AND SOCIAL RESILIENCE

The COVID‐19 pandemic and its containment measures have had a profound impact on the economy and society, with existing literature addressing four primary areas: macroeconomics, supply of essential goods, vulnerable populations, and mental health. China has implemented targeted measures in each of these areas.

### 6.1 Macroeconomics

The concern that the pandemic would lead to economic recession is indisputable [122, 123], but researchers differed on the correlation between the stringency of COVID‐19 measures and losses in gross domestic product (GDP) [124]. Some studies based on production or equilibrium models have estimated the direct effects of COVID‐19 shutdowns on macroeconomics, suggesting that tougher COVID‐19 measures and lockdown would cause greater GDP losses [125, 126, 127]. However, by comparing the macroeconomic outcomes of countries that adopted different COVID‐19 policies, no significant trade‐off was found between restrictive policies and GDP [128]. Furthermore, when including the costs of infection in cost–benefit analyses, countries adopting stricter containment strategies have shown a less severe effect on GDP growth [129, 130].

China prioritized economic recovery while implementing pandemic prevention and control measures. Early in the COVID‐19 epidemic, enterprises producing critical pandemic‐related materials were encouraged to resume production [86, 131]. Since April 2020, virus‐specific guidelines for various industries and public settings were issued, facilitating the safe resumption of normal activities [132]. Support was also provided to small and medium‐sized enterprises to stimulate consumption [133] through tax reductions, financial credit, and insurance schemes [134, 135, 136]. As a result, China's economy grew by 2.3% in 2020, making it the only major economy to maintain positive growth [137]. In 2021, China's GDP grew by 8.4% [138], with its import and export scale reaching a record USD 6.05 trillion, a year‐on‐year increase of 21.4% [139]. In this way, the experience of China offered a new perspective on the interaction between public health measures and macroeconomics.

### 6.2 Supply of essential goods

The availability of crucial medical supplies and daily necessities received widespread attention. The outbreak caused a sudden increase in the need for masks, ventilators, and other medical products [140, 141]. Many countries encountered extreme shortages of personal protective equipment (PPE), even in developed countries such as the US [142], the UK [143], and Japan [144]. Disruptions in the PPE supply chain and the uncontrolled distribution of inventories were the main reasons behind these shortages [145, 146]. Beyond PPE, the food supply also raised concerns about food insecurity caused by unemployment, poverty, and the breakdown of food production and distribution chains during the pandemic [147, 148, 149, 150].

By organizing the resumption of production in industries that manufactured critical medical supplies and taking full control over production and distribution [131, 134], a national distribution platform was established by the Chinese government to ensure the rapid, orderly use of these supplies, prioritizing key regions, key operations, and key patients, especially those with severe illness [151]. On a social level, many state‐owned and private enterprises offered to shift their focus to produce medical supplies and protective equipment [152, 153, 154]. Through these efforts, China was able to produce 3.4 million medical N95 masks and 1.5 million sets of disposable protective suits daily [4]. To ensure the availability of daily necessities such as grain, vegetables, and meat during lockdowns, governments set up “Green Channel” and e‐commerce platforms to secure these supplies [155, 156, 157, 158, 159]. For individuals under at‐home quarantine, community‐appointed personnel regularly delivered daily necessities [160].

### 6.3 Vulnerable populations

The working and living conditions of frontline health workers and other vulnerable groups in society have also been widely discussed. Great challenges such as adapting to changes in disease progression, facing a high risk of infection with limited protective equipment, and burnout owing to extended working hours made frontline health workers a particularly vulnerable group during the pandemic [161, 162, 163]. Similarly, elderly adults, children, disabled people, and other disadvantaged subgroups also faced higher risks of infection and struggled to survive during the pandemic, with their health and livelihood negatively affected [164, 165, 166].

China enacted a set of measures to address the above issues [167]. Except for the priority allocation of PPE, the government requested all units to fully support daily services for medical personnel, such as providing good conditions for adequate rest, essential drugs, and nutritious dining services. Civil affairs departments and communities arranged temporary or long‐term living care for elderly and disabled people as well as minors lacking guardianship owing to the pandemic [168]. Timely assistance, such as temporary accommodation and food, was provided to those stranded as a result of traffic and mobility controls [169].

### 6.4 Mental health

As a global health emergency, COVID‐19 also led to a mental health crisis. The adverse psychological impacts related to COVID‐19, such as anxiety and depression disorders, sleep disorders, increased suicide risk, and posttraumatic stress disorder (PTSD), which are likely to be associated with physical symptoms, became more prevalent in the general population [170, 171, 172, 173]. Social distancing, economic hardship, health risks, discrimination, and stigmatization are considered the main factors contributing to these adverse psychological effects [174, 175]. Many intervention measures have been proposed, such as providing resources for psychological support, timely updating of evidence‐based information, and reducing loneliness using online platforms [174, 175].

In comparison with other countries, good acceptance of mask‐wearing and hand hygiene, experiencing fewer physical symptoms, and greater access to health information in China led to lower levels of anxiety and depression [176, 177, 178]. To alleviate post‐traumatic stress disorder (PTSD) among the general population, all provinces in China offered a 24‐h psychological assistance hotline that could provide free psychological counseling, especially for health workers. Additionally, timely and effective provision of health information online [179], including webcasts showing the construction of makeshift hospitals, helped many Chinese people overcome the quarantine period and feel positive about the actions of the government, greatly alleviating psychological pressure among all residents of China.

## 7 CHINA'S ENGAGEMENT IN GLOBAL HEALTH

Health is an area where there is broad consensus on global priorities and a potentially fertile space for building new forms of cooperation [180]. China actively participates in the work of the WHO and other international organizations in the field of health, takes the initiative to share best practices in China's health development, and firmly adhering to the concept of community with a shared destiny [181, 182]. As a responsible power in world affairs, developing the capacity to exert effective global health governance is imperative [183]. Given this challenge, China initiated a strong global emergency humanitarian response focused on prioritizing human life and fostering a global health community [184, 185].

### 7.1 Knowledge‐sharing and information exchange

The Chinese government places great emphasis on international dialogue [186] and is highly committed to global pandemic prevention efforts [187]. As summarized in the *Timeline on COVID‐19 Information Sharing and International Cooperation* [188], China quickly informed the WHO about the COVID‐19 outbreak, identified the causative pathogen [189], released autopsy results of infected individuals, and shared the virus's genome sequence [190]. Diagnostic and treatment guidelines were subsequently published, translated, and commended by the WHO [191].

An academic sharing platform facilitated nationwide research into the origin of SARS‐CoV‐2, clinical treatments, and testing methods [192]. China's comprehensive reporting of drug screening results and essential treatment techniques fostered global information sharing [193]. Universities, hospitals, Chinese citizens overseas, and international students actively disseminated preventive measures [159], encouraging many countries to adopt stricter public health strategies [194, 195] (Table 3).

**Table 3:** International assistance initiated by China during the COVID‐19 pandemic.

China's global engagement		Receiver	Quantity
Financial assistance			
		WHO	50 million dollars
		COVID‐19 Vaccines Global Access (COVAX)	100 million dollars
Supply assistance			
	Protective suits	153 Countries	4.6 billion
	Face masks	15+	430 billion
	Testing Reagents	Intl. Organizations	18 billion
	Doses of vaccine	120+ Countries	More than 2.2 billion
		& Intl. Organizations	
Medical support			
	COVID‐19 Expert groups	34 Countries	38 groups
	Introduce the diagnosis & treatment plan of traditional Chinese medicine	150+	Depends on their demand
	Provide Chinese medicine products	Countries & Regions	
Knowledge sharing			
	Training courses	56 Countries	400+
	Exchanging views at high level	180+ Countries & Regions,	50+
	Technical exchange activities	10+ Intl. Organizations	300+

*Source*: Seventy‐Sixth World Health Assembly held in Geneva, Switzerland, from May 21 to 30, 2023 (2023 marks the 60th anniversary of the dispatch of Chinese medical teams to foreign countries).

Abbreviation: WHO, World Health Organization.

### 7.2 Bilateral assistance and multilateral supply

Initially receiving international support, China later became a crucial global supplier of pandemic prevention materials [185], demonstrating a generous and selfless spirit of international cooperation [196]. Policies, management manuals related to the construction of fangcang hospitals, and clinical guidelines were translated, and medical expert teams were dispatched to 34 countries [197, 198]. By learning from the experience in China, other countries could identify appropriate methods to control the pandemic [199, 200].

In May 2021, China's COVID‐19 vaccine was included in the Emergency Use Listing. The Chinese government pledged to make the COVID‐19 vaccine available on the basis of the global public good and to cooperate with developing countries in vaccine production and supply [201]. China has also promoted TCM via more than 100 live online events, introducing prevention and treatment solutions to more than 150 countries and regions.

China supports international organizations such as the United Nations, WHO, African Union, and World Food Program, assisting in fundraising, procurement, and stockpiling [202]. Public–private partnerships are encouraged, mobilizing domestic and international organizations to provide material supplies, online forums, and volunteer support (Table 3).

## 8 DISCUSSION

In the face of a global pandemic, China's robust centralized approach provides a template for rapid response, demonstrating the potential benefits of a centralized command in managing large‐scale health crises. This structure is particularly suitable for a nation like China, where swift execution is urgently needed across vast territories and diverse populations [203].

Evidence from countries such as China [204, 205] and others [206, 207, 208] suggests that mass quarantine and mobility control measures can significantly inhibit the spread of infectious diseases like COVID‐19. Although implementation was not without challenges, especially during large migratory events, the nation's agility in health infrastructure scalability was evident. The expedited construction of fangcang hospitals [196], fast implementation of sophisticated quarantine measures [209], and rapid deployment of a mass testing infrastructure [210, 211] not only showcased China's proactive response to soaring case numbers but also provided a model that could be replicated in crises where health care systems face imminent overburdening.

The economic and societal resilience exhibited by China proved central to its pandemic control success. Often referred to as the “world's factory,” China demonstrated its industrial and economic resilience by maintaining production of essential medical and food supplies that have withstood the most difficult periods during the pandemic [212, 213, 214, 215]. Simultaneously, the challenges faced by vulnerable populations highlight the need for holistic crisis management, emphasizing individuals' physiological and psychological well‐being [216, 217, 218].

In the realm of global engagement, China's prompt release of the SARS‐CoV‐2 genome sequence, coupled with the dissemination of effective practices, accentuated its central role in international health diplomacy [188]. These efforts facilitated collaborative research and paved the way for global countermeasures. Over the course of the pandemic, digital innovations have empowered pandemic responses, although these have also raised concerns about user privacy and the balance between surveillance and public health safety [219]. In the future, more digital tools, ranging from contact tracing to AI‐driven analytics, could be used within and across nations, with requisite caution.

## 9 CONCLUSION

China's holistic approach to the COVID‐19 crisis involved a comprehensive, multi‐pronged strategy that ranged from swift mobilization of emergency resources to adaptive and scientifically supported disease prevention measures. Balanced socioeconomic initiatives ensured both public well‐being and economic stability. Furthermore, China's unwavering commitment to fostering global health collaboration showcased its dedication to a united, international effort against the pandemic. For enhanced global health security, nations can draw insights from China's approach, adapting it to their unique context. Continuous research can refine these strategies to address future health challenges globally.

## AUTHOR CONTRIBUTIONS

**You Wu**: Conceptualization (equal); methodology (equal); project administration (equal); writing—original draft (equal); writing—review and editing (equal). **Zijian Cao**: Visualization (lead); writing—original draft (equal); writing—review and editing (equal). **Jing Yang**: Writing—original draft (equal). **Xinran Bi**: Writing—original draft (equal). **Weiqing Xiong**: Writing—original draft (equal). **Xiaoru Feng**: Writing—original draft (equal). **Yue Yan**: Writing—original draft (equal). **Zeyu Zhang**: Writing—review and editing (equal). **Zongjiu Zhang**: Conceptualization (equal); funding acquisition (equal); project administration (equal); writing—review and editing (equal).

## CONFLICT OF INTEREST STATEMENT

Professor Zongjiu Zhang is the member of the *Health Care Science* Editorial Board. To minimize bias, he was excluded from all editorial decision‐making related to the acceptance of this article for publication. The remaining authors declare no conflict of interest.

## ETHICS STATEMENT

Not applicable.

## INFORMED CONSENT

Not applicable.

## Data Availability

Data sharing is not applicable to this article as no new data were created or analyzed.

## 1

**Table A1:** Laws and regulations related to infectious disease prevention and control in The People's Republic of China (PRC).

	Title	Enactment Date
Law	Law of the PRC on the Prevention and Treatment of Infectious Diseases	1989/9/1
	Law of the PRC on Public Security Administration Punishments (excerpt)	1987/1/1
	Law of the PRC on Wildlife Protection (excerpt)	1989/3/1
	Law of the PRC on Criminal Offenses (excerpt)	1997/10/1
	Law of the PRC on Emergency Response	2007/11/1
	Law of the PRC on Basic Healthcare and Health Promotion	2020/6/1
	Law of the PRC on Animal Epidemic Prevention (excerpt)	2021/5/1
Administrative regulations	Implementation Measures of the Law of the PRC on Prevention and Treatment of Infectious Diseases	1991/12/6
	Implementation Rules of the Law of the PRC on Border Health Quarantine	1989/3/6
	Regulations on Hygiene Management of Public Places	1987/4/1
	Regulations on Health Quarantine for Domestic Transport	1999/3/1
	Regulations on the Emergency Response to Public Health Emergencies	2003/5/9
	Regulations on the Emergency Response to Major Animal Epidemics	2005/11/16

**Appendix—method**

In literature search, Boolean operators were used to combine key terms tailored to each domain and subcategory, like “surveillance,” “border control,” “public health interventions,” and “vaccination,” with the term “COVID‐19” to ensure relevance. Additional keywords or phrases identified in these abstracts were used to refine and expand the search. Here is a list of the refined keyword strategy for each domain.

1. **Theoretical Framework:**
  - Keywords:
    ∘ (“emergency management” OR “crisis management” OR “disaster response”) AND (“theory” OR “model” OR “framework”) AND “pandemic”
2. **National Infrastructure**:
  - Keywords:
    ∘ (“government structure” OR “health system” OR “health policy” OR “legislation” OR “public engagement” OR “emergency law”) AND (“pandemic” OR “COVID‐19”)
3. **Medical Response**:
  *3.1 **Systems & Mechanisms:**
    - Keywords:
      ∘ (“medical response” OR “emergency medical system” OR “medical personnel deployment” OR “military support”) AND (“pandemic” OR “COVID‐19”)
  *3.2 **Evidence‐based Treatment:**
    - Keywords:
      ∘ (“treatment guidelines” OR “clinical management” OR “viral evolution”) AND (“pandemic” OR “COVID‐19”)
  *3.3 **Scientific Research:**
    - Keywords:
      ∘ (“treatment protocol” OR “medication development” OR “vaccine development” OR “clinical trial”) AND (“COVID‐19”)
4. **Prevention and Control Strategy**:
  *4.1 **Surveillance & Monitoring:**
    - Keywords:
      ∘ (“surveillance” OR “monitoring” OR “mass testing” OR “testing strategy” OR “case detection”) AND (“COVID‐19”)
  *4.2 **Border & Flow Control:**
    - Keywords:
      ∘ (“border control” OR “travel restriction” OR “quarantine policy” OR “isolation guidelines”) AND (“COVID‐19”)
  *4.3 **Public Health Interventions:**
    - Keywords:
      ∘ (“public health interventions” OR “masking” OR “social distancing” OR “hygiene promotion”) AND (“COVID‐19”)
  *4.4 **Vaccination:**
    - Keywords:
      ∘ (“vaccination” OR “immunization” OR “vaccine distribution” OR “rollout plan” OR “vaccine allocation”) AND (“COVID‐19”)
5. **International IT Advances and COVID‐19 Response**:
  *5.1 **Contact Tracing:**
    - Keywords:
      ∘ (“contact tracing” OR “digital tracking”) AND (“COVID‐19”)
  *5.2 **Health Code:**
    - Keywords:
      ∘ (“health code” OR “QR code” OR “digital pass”) AND (“COVID‐19”)
  *5.3 **Data Security:**
    - Keywords:
      ∘ (“data privacy” OR “data security” OR “ethical concerns”) AND (“tech solutions” OR “contact tracing” OR “health code”) AND (“COVID‐19”)
6. **Socioeconomic Factors**:
  *6.1 **Economic Development & Society:**
    - Keywords:
      ∘ (“economic impact” OR “society disruption” OR “economic recovery” OR “employment” OR “job opportunity” OR “tax reductions” OR “financial credit support” OR “insurance scheme” OR “interest compensation”) AND (“COVID‐19”)
  *6.2 **Supply:**
    - Keywords:
      ∘ (“food supply” OR “goods supply” OR “medical supply chain”) AND (“COVID‐19”)
  *6.3 **Protecting Vulnerable People:**
    - Keywords:
      ∘ (“vulnerable population” OR “elderly” OR “children” OR “disabled”) AND (“protection” OR “support”) AND (“COVID‐19”)
  *6.4 **Mental Wellbeing:**
    - Keywords:
      ∘ (“mental health” OR “wellbeing” OR “psychological impact”) AND (“pandemic” OR “COVID‐19”)
7. **Global Engagement**:
  - Keywords:
    ∘ (“global cooperation” OR “international collaboration” OR “global response”) AND (“COVID‐19”)
    ∘ (“knowledge sharing” OR “information exchange” OR “best practices sharing”) AND (“pandemic” OR “COVID‐19”)
    ∘ (“bilateral assistance” OR “multilateral support” OR “aid distribution” OR “global supply chain”) AND (“COVID‐19”)
